# Chemical Visualization of a GaN p-n junction by XPS

**DOI:** 10.1038/srep14091

**Published:** 2015-09-11

**Authors:** Deniz Caliskan, Hikmet Sezen, Ekmel Ozbay, Sefik Suzer

**Affiliations:** 1 Nanotechnology Research Center, Department of Electrical and Electronics Engineering and Department of Physics, Bilkent University, 06800, Ankara, Turkey; 2 Department of Chemistry, Bilkent University, 06800 Ankara, Turkey

## Abstract

We report on an operando XPS investigation of a GaN diode, by recording the Ga2p_3/2_ peak position under both forward and reverse bias. Areal maps of the peak positions under reverse bias are completely decoupled with respect to doped regions and allow a novel chemical visualization of the p-n junction in a 2-D fashion. Other electrical properties of the device, such as leakage current, resistivity of the domains are also tapped via recording line-scan spectra. Application of a triangular voltage excitation enables probing photoresponse of the device.

The diode or the p-n junction is the single most important functional and central element of almost all of the electronic and optoelectronic devices^1^. Therefore, its design, fabrication as well as characterization are all important issues. Traditionally, elaborate but mostly electrical based techniques have been utilized for detection and/or characterization of a device which usually includes a number of different electrical units, such as junctions, transistors, etc. On the other hand, powerful analytical techniques are increasingly being used for probing chemical and physical together with electrical properties of materials and devices in much smaller size and under realistic operating conditions. Scanning Electron microscopy (SEM) is one of these methods, where imaging with submicron lateral resolution is an added advantage. This was first shown by Chang and Nixon reporting on observation of a significant change in the secondary electron yield across a p-n junction^2^. Kaetsner *et al*.^3^ used SEM to demonstrate a direct observation of the potential variations inside a 90-nm GaAs channel^4^, and Li *et al*. in carbon nanotubes^5^. Electrical biasing together with SEM mapping is a recent elaboration of the technique providing direct imaging of the p-n junction, including its depletion width, made up from GaInP/GaAs^4^ heterojunctions, and in GaN nanowires^5^,^6^,^7^. Cathodoluminescence in a scanning transmission microscope (CL-STEM)^8^ and conductive atomic force microscopy are other techniques used to characterize the p-n junction in GaN nanorods^9^ and porous GaN based LEDs^10^. Formation and characterization of p-n junctions in photodiodes and photovoltaics have also been heavily investigated using a combination of electrical and optical analyses tools^10^,^11^,^12^,^13^,^14^,^15^,^16^,^17^.

However, all of these techniques provide very limited chemical information, if any. In this respect, electron spectroscopic techniques, such as Auger Electron Spectroscopy (AES) and X-Ray Photoelectron Spectroscopy (XPS) have the ability to reflect the electrical potential of the medium surrounding the probed atom, created intentionally or not^18^,^19^,^20^,^21^,^22^,^23^,^24^,^25^,^26^,^27^,^28^,^29^,^30^,^31^, and render them even more powerful for investigating materials and especially device performances under operational conditions, the so-named in-operando AES or XPS. Use of AES for probing the electrical potential variations across a working device was first reported three decades ago, but the technique has not been pursued due to its limited chemical specificity^32^,^33^,^34^,^35^,^36^. Numerous successful reports have also appeared for probing potential variations across a p-n junction device using PEEM (photoemission electron microscopy), a variant of electron spectroscopy, but again it also has limited chemical specificity^31^,^37^,^38^,^39^,^40^,^41^. Exciting applications towards investigating devices under more realistic operational conditions are now being reported as a consequence of the recent advances in ambient-pressure XPS (APXPS)^42^,^43^,^44^,^45^. In parallel, recent advances in commercial XPS instrumentation, like micro-focusing and parallel detection systems, have also provided new possibilities to record XPS data with higher lateral resolution and good statistics in reasonable measurement times^46^.

In one of our recent publications, we presented an XPS investigation of a CdS-based photoresistor, taking advantage of these instrumentation capabilities, and under working conditions for the device^47^. The electrical potential variations across the device were mapped by recording the binding energy positions under an applied +6 V d.c. bias across the electrodes, and with and without laser illumination at different wavelengths. Variations in the Cd3d_5/2_ peak positions were used to extract electrical parameters and detect the presence of morphological defects affecting the performance of the device in a chemically specific fashion. A similar study was also presented for a CVD grown resistive graphene layer between two gold electrodes, and by imposing current flow through the device. Such a procedure allowed us to detect the presence of morphological defects and cracks when the graphene layer was subjected to a mild oxidation^48^. Furthering that study, we also reported on an investigation where gate-tunable photoemission was recorded of a graphene transistor, for the graphene as well as the Si_3_N_4_ substrate layers^49^. Another one of our publication reported investigation of the p-n junction of a commercial Si-diode during its operation under forward and reverse bias^50^. The present work extends such an investigation to the entire surface (2 dimensional) of a GaN device consisting of three parallel diodes, as shown in Fig. 1a, but only one is operated at a time. GaN is a wide band-gap compound semiconductor with superior electronic and optoelectronic properties utilized in numerous devices for light emitting diodes, sensors and transistors for high power and high frequency applications^51^. Therefore, better understanding of physical and chemical properties of devices based on GaN are expected to pave ways to technological advancement in numerous applications.

## Results

**Figure 1:**
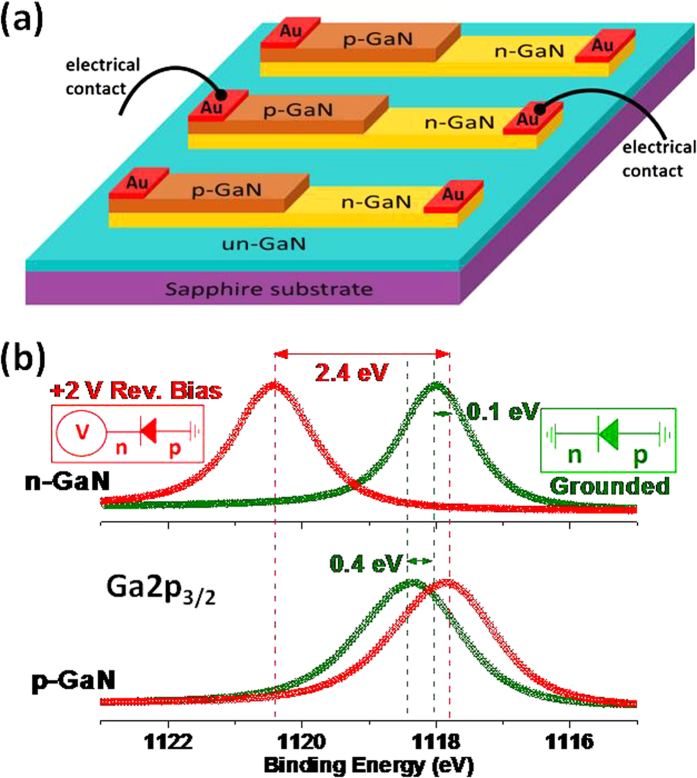
(**a**) Schematic of the device and its electrical contacts. (**b**) XPS spectra. Ga 2p_3/2_ peak recorded under 0 and +2 V Reverse Bias in the p and n regions. The insets show wirings of the electric circuit.

Figure 1b depicts the Ga 2p_3/2_ peak of the p- and n- regions of the middle device recorded under +2 V bias as well as when grounded. For an undoped GaN (un-GaN), the tabulated binding energy of the Ga2p_3/2_ is 1117.9 eV, there is already a small binding energy difference of 0.4 eV between p- and n- regions when the XPS data is recorded in the conventional (both electrodes grounded) fashion, due to differences in their Femi-level pinning at the surface, in accordance with published data^51^,^52^,^53^,^54^,^55^, which was discussed in detail in our previous work^56^,^57^. The more dramatic change between the p- and n- regions materializes when recorded under +2 V reverse bias, upon which the peaks in the n- and p-regions exhibit 2.4 eV relative binding energy difference, due to charge built-up across the junction. We have also measured the shift in the Au4f_7.2_ peak of the gold electrodes connected to yield only 2.02 eV reproducing faithfully the bias voltage within the experimental uncertainty of 50 meV. This finding validates the accuracy and the precision of our measurements, and also confirms the absence of any significant contact resistance(s) within the entire experimental set-up. The persistence of the additional and opposite sign 0.4 eV difference between the n- and p-regions, even under 2 V bias is remarkable.

**Figure 2:**
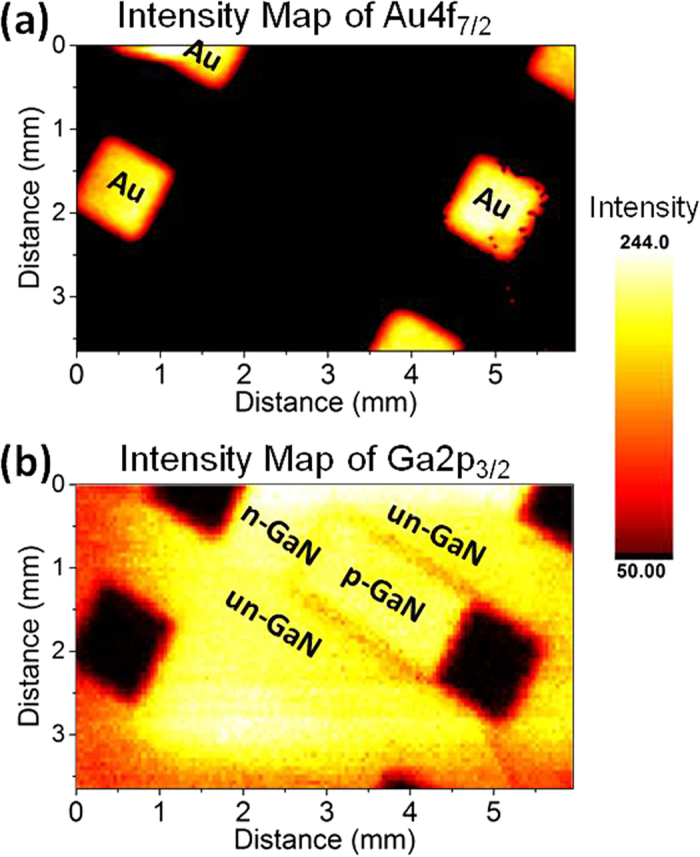
XPS Intensity Maps. Areal maps of the intensity of the Au4f_7/2_ and Ga2p_3/2_ peaks derived from peak areas.

**Figure 3:**
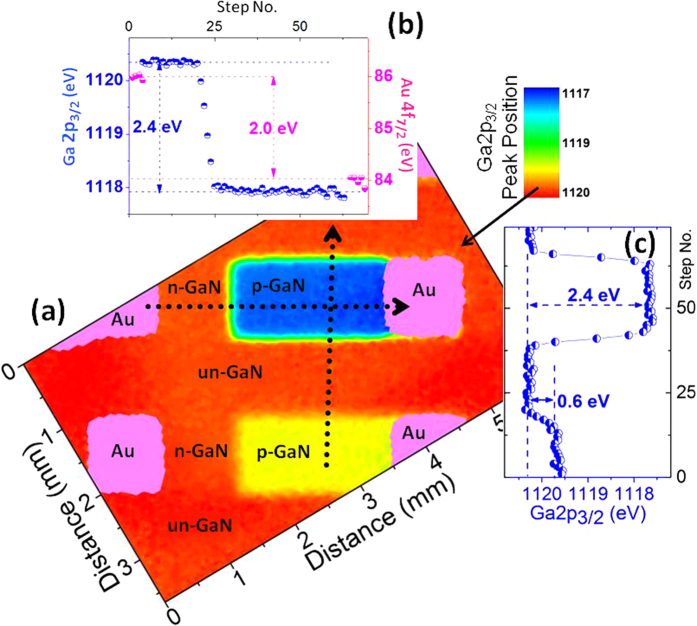
XPS Binding Energy Map. (**a**) Areal map of the variations in the binding energy of Ga2p_3/2_ recorded under +2 V reverse bias. (**b**) The peak positions of Au4f_7/2_ and Ga2p_3/2_ peaks along the lines shown. (**c**) The peak positions of Ga2p_3/2_ peak traversing the p-n junction in a perpendicular direction.

Figure 2 displays areal intensity maps of the Au4f_7.2_ and the Ga2p_3/2_ peaks recorded in the snap-shot mode. The intensity variations of the Ga peak between various regions are indistinguishable except at the intersection of the un-GaN and the p-GaN regions. In Fig. 3, the areal map of the measured binding energy positions of the Ga2p_3/2_ peak under +2 V reverse bias is displayed, where now the differences are transformed into the voltage space and amplified. As shown in the inset, the two doped regions display 2.4 eV difference in the binding energy position under +2 V reverse bias, traversing across the gold electrodes. Traversing in the middle and perpendicularly crosses the side-walls of the intersection of the un-GaN and the grounded p-GaN twice. Interestingly although an equivalent of the full junction potential of 2.4 eV is measurable at the side-walls of the un- and p- intersection (see Fig. 3b), only 0.6 eV voltage drop is measured between the un- and the floating p- regions of the neighboring diode (Fig. 3c), indicative of a certain cross-talk among the diodes. Unfortunately, our spatial resolution of >50 μm does not permit assessing the depletion length or other similar properties of the junction^4^,^5^, except for stating that the depletion length is smaller than 50 μm. Note also that the measured binding energy difference in the Au4f_7.2_ peak of the two gold electrodes is still consistently 2.0 eV, as is also indicated in the inset, and we reiterate that each GaN region and their interfaces exhibit different binding energy shifts.

**Figure 4:**
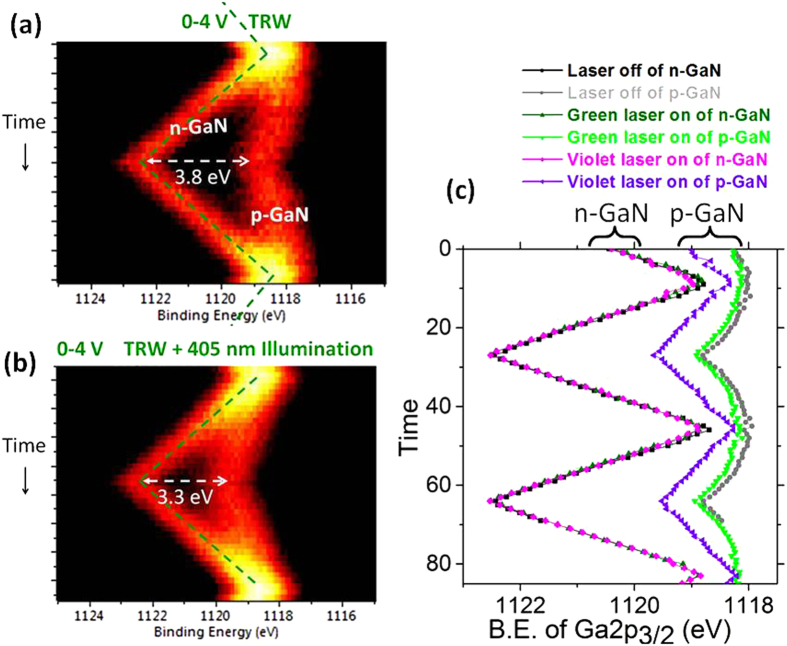
XPS Measuerements under 0–4 V Triangular Bias. Ga2p_3/2_ peak recorded under 0–4 V triangular excitation at the junction. (**a**) Without illumination. (**b**) Under Viloet Light Illumination. (**c**) Derived peaks positions.

**Figure 5:**
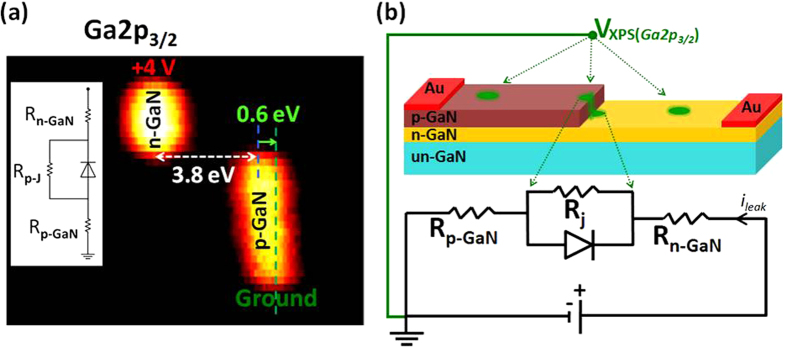
XPS line scans. (**a**) Ga2p_3/2_ peak recorded across the junction in the line scan mode under +4 Reverse Bias. (**b**) The equivalent circuit model.

Further XPS analyses of the junction were carried out by imposing a slow time-varying triangular voltage stimuli of 0–4 V, and also by exposing the sample to light illumination with different colors. In Fig. 4a, the time variations of the Ga2p_3/2_ peak of a 200 μm spot is shown, where the spot is positioned on the junction covering both the n- and p- sides almost equally. When the device is forward biased, the Ga2p_3/2_ peaks of the n- and p-regions overlap, but start splitting apart into two components when the device enters the rectifying mode. The splitting increases up to the turning point with a reproducible symmetry. Note, however, the rectification is not all that perfect and small but a measurable slope is also displayed by the p-component, due to unwanted leakage current, which is also present in the I–V characteristics of the device, as shown in the Supplementary Information***(SI) section*** as Figure S1. In addition, as shown in Fig. 4b,c, whereas illumination by green laser does not alter the performance of the p-n junction, the performance is degraded further under violet light illumination, which leads us to postulate the presence of a parallel resistor (R_p_) controlled by violet light, i.e. a photoresistor. A simple equivalent circuit can now be formulated, which is given in Fig. 5 and also in the ***SI section***, by assuming the presence of two series resistor in the n- and p- regions and a parallel (photo) resistor across the junction, in order to account for this realistic performance of the device, at least for its d.c. and/or low frequency response^11^. In principle both the green 532 nm (2.3 eV) and the violet 405 nm light (3.1 eV) should not have contributed to the band to band photoconductivity of GaN with a band-gap of 3.4 eV^54^. Hence, the presence of deep traps and defects must be causing such changes, which was also reported in our previous studies^56^,^57^.

Consistency and symmetry checks of our analyses are also performed by recording normal scanned spectra in the form of line-scans, with higher energy resolution and better accuracy as shown in Fig. 5a across the gold electrodes, by application of +4 and −4 V reverse bias geometries (as shown in Figure S2 of the ***SI section***), and recording both the Ga2p_3/2_ as well as the Au4f_7.2_ peaks (not shown). Going across the device a total of 4.4 eV voltage drop is measured in the Ga_3/2_ peak, whereas only 4.0 eV in the Au one, consistent with the persistent 0.4 eV Fermi-level difference between the n- and p-GaN regions. In accordance with the results of the triangular excitation, only 3.8 V of the applied reverse bias develops across the junction, and the remaining 0.6 V through the p-region only. Knowing that the p-GaN is more resistive^56^,^57^, we can estimate the value of the series resistor (R_p_) as ~0.9 kΩ, in the p-region, and an almost negligible one for the n-one, using the equivalent circuit model, and the 700 μA leakage current extracted from Figure S1 of ***the SI section***. Noting that under violet light illumination, the voltage drop across the junction drops down to 3.3 eV (see Fig. 4b,c), and assuming that the R_p_ does not change significantly, we can now estimate that an additional ~580 μA leakage current (totaling to ~1.28 mA) is introduced by the violet light illumination. Details of the computations are given in the ***SI section***. Admittedly, this is a grossly oversimplified approach, since band-bending and other processes, impurities, defects, etc., are expected to contribute significantly to the overall voltage variations in a working device. On the other hand, our strategy offers a pivotal experimental tool for sorting out such effects, and calls for further experimental as well as theoretical and/or modeling work.

In summary, many of the electrical properties of a p-n junction can be probed and visualized in a chemically specific fashion during its realistic operating conditions (in-operando) by recording XPS data, while imposing different forms of voltage and optical stimuli. This simple and novel experimental methodology opens up completely new avenues for utilization of the 5-decades old but powerful XPS technique for chemically specific electrical properties of both materials and devices with respect to the otherwise impossible performance- and/or failure-analyses.

## Methods

### Sample Preparation and Device Fabrication

The epitaxial structure growth was started with desorption cleaning of C plane sapphire substrate at 1100 °C under H_2_ flow. Low temperature AlN nucleation layer was grown at 50 mbar reactor pressure and 790 °C substrate temperature. Following the growth of 700 nm thick ud-GaN layer at 200 mbar and 1050 °C SiH_4_ source was opened at the same growth condition to provide n- type doping. After 750 nm growth n-GaN layer temperature decreased to 980 °C and Cp_2_Mg was introduced to the reactor for p- doping of 350 nm thick p-GaN layer. Following the growth, wafer was annealed at 800 °C for 10 min in order to activate Mg impurities. Device fabrication was started with photolitograhy for p-GaN mesa etching. Etching process was performed with 800 W ICP power and 100 W RF power at 0.4 Pa reactor pressure using BCl_3_ (20 sccm)/Cl_2_ (25 sccm)/Ar (10 sccm) gases. After 700 nm etching a second photolitograhy is performed for a wider area of device mesa and etched for a total etch dept of 1.4 μm, reaching the ud-GaN layer. Ti (15 nm)/Al (40 nm)/Ni (40 nm)/Au (40 nm) metals were e-beam deposited on photolitographically defined n-type ohmic contacts and annealed at 850 °C for 30 s in the forming gas atmosphere. Similarly Ni (15 nm)/Au (1500 nm) metals were used to form p-type ohmic contacts and annealed at 800 °C for 30 s. The I–V characteristics of the fabricated device is shown in Figure S1 of ***the SI section.***

### XPS Measurements

A Thermo Fisher K-Alpha electron spectrometer with monochromatic AlKα X-rays was used for the XPS analyses. The instrument is modified to allow imposition of an external voltage bias across the sample during data acquisition^47^,^48^,^49^,^50^. For normal scans an X-ray beam size of 400 μm was used, but for the rest of the data presented in this work a 50 μm-diameter X-ray spot size was used both in recording the mapped regions as well as the line scans with 50 μm steps in between data points. A 50 mW violet at 405 nm (3.1 eV) and green at 532 nm (2.3 eV) solid states lasers in continuous wave mode are used for photoillumination.

## Additional Information

**How to cite this article**: Caliskan, D. *et al*. Chemical Visualization of a GaN p-n junction by XPS. *Sci. Rep*. **5**, 14091; doi: 10.1038/srep14091 (2015).

## Supplementary Material

Supplementary Information
