# Supplementary material for: Chemical Visualization of a GaN p-n junction by XPS
Source: Sci Rep. 2015 Sep 11;5:14091. doi: 10.1038/srep14091 (PMC4566124; doi:10.1038/srep14091)
Supplement: Supplementary Information [file srep14091-s1.pdf]

SUPPLEMENTARY INFORMATION

Chemical Visualization of a GaN p-n junction by XPS

Deniz Caliskan,[a] Hikmet Sezen,[b] Ekmel Ozbay,[a] Sefik Suzer*,[b]

[a]Nanotechnology Research Center, Department of Electrical and Electronics Engineering and Department of Physics, Bilkent University, 06800, Ankara, Turkey

[b]Department of Chemistry, Bilkent University, 06800 Ankara, Turkey

*Corresponding author (suzer@fen.bilkent.edu.tr)

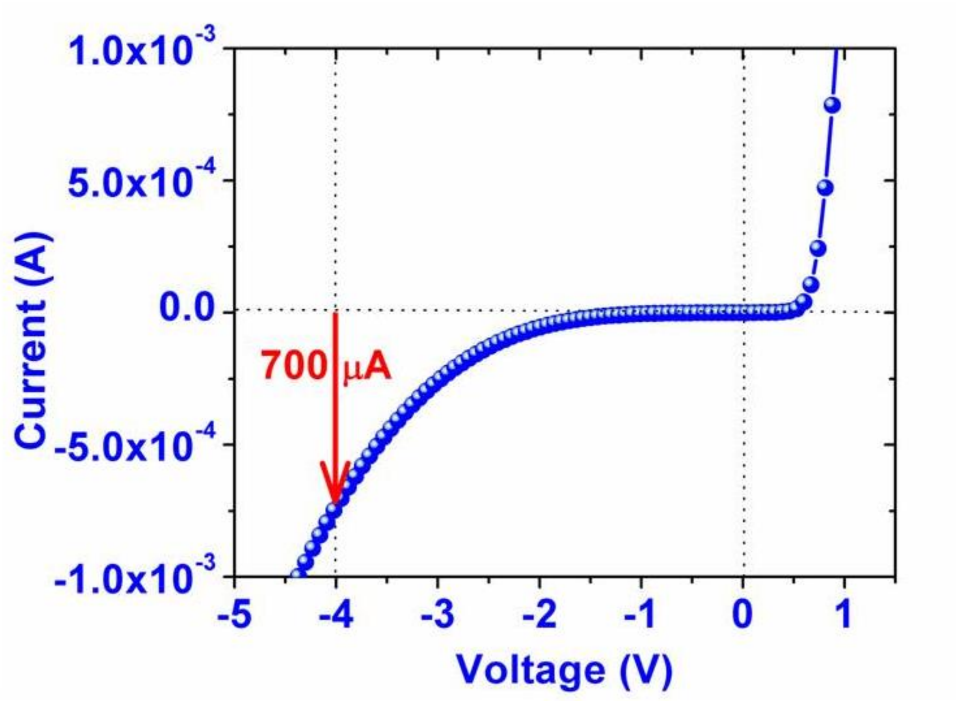

Figure S1. Measured I-V curve of the middle device.

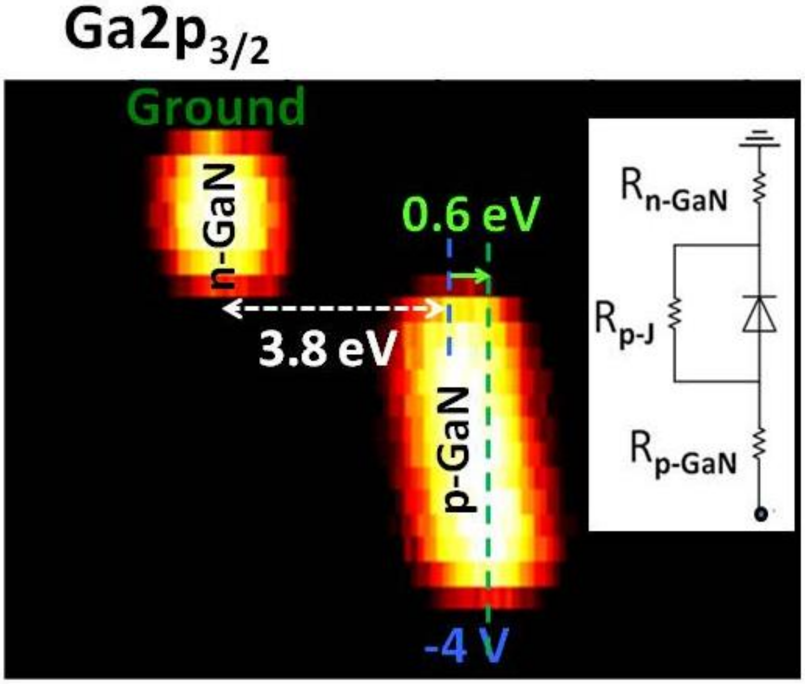

Figure S2. XPS line scans. Ga2p$_{3/2}$ peak recorded across the junction in the line scan mode under -4 V Reverse Bias and the equivalent circuit model.

Equivalent Circuit and Computations

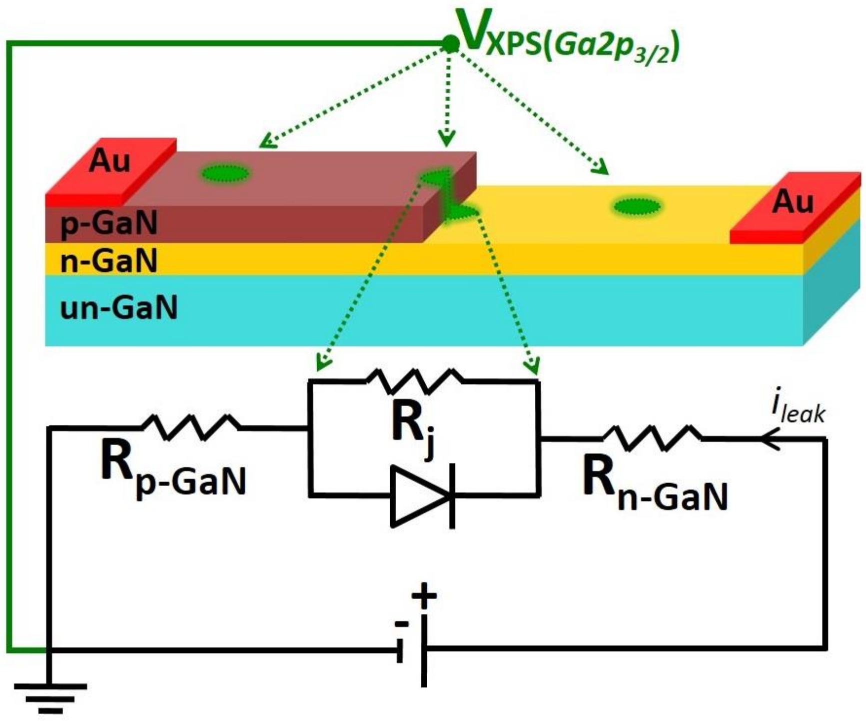

NO ILLUMINATION

I (overall) = 0.7 mA (see Figure S1)

$4.0\ V = I * R = 0.7\ x10^{-3}\ A * R_T \rightarrow \quad R_T = 5.7\ k\Omega \qquad R_T = R_n + R_j + R_p$

IR drop [p-region, Figure 5a] $= 0.6\ V = 0.7\ x10^{-3}\ A * R_p \rightarrow R_p = 0.86\ k\Omega$

Assuming R_n to be negligible $\rightarrow R_j = 4.8\ k\Omega$

UNDER 405 nm ILLUMINATION

Assuming R_p does not change i.e. $R_p = 0.86\ k\Omega$

IR drop [p-region, Figure 4a,c] $= 1.1\ V = I_{photo}$ (overall) $* 0.86\ k\Omega \rightarrow I_{ph} = 1.28\ mA$

$4.0\ V = I * R = 1.28\ x10^{-3} A * R_T(\text{photo}) \rightarrow R_T(\text{photo}) = 3.1\ k\Omega$ and $R_j(\text{photo}) = 2.3 k\Omega$
